# Pretreatment and dynamic neutrophil-to-lymphocyte ratio in relation to CT-based volumetric response to platinum-based neoadjuvant chemotherapy in NSCLC

**DOI:** 10.17305/bb.2026.14043

**Published:** 2026-04-22

**Authors:** Sinisa Maksimovic, Ivan Kuhajda, Jelena Djekic-Malbasa, Dragana Tegeltija, Tamara Maksimovic, Ivan Ergelasev

**Affiliations:** 1 Faculty of Medicine, University of Novi Sad, Novi Sad, Serbia; 2 Institute for Pulmonary Diseases of Vojvodina, Sremska Kamenica, Serbia; 3 Oncology Institute of Vojvodina, Sremska Kamenica, Serbia

**Keywords:** Non-small cell lung cancer, neutrophil to lymphocyte ratio, neoadjuvant chemotherapy, tumor volumetry, treatment response.

## Abstract

The neutrophil to lymphocyte ratio (NLR) serves as a marker of systemic inflammation and is an established prognostic factor in non-small cell lung cancer (NSCLC). However, its predictive value for treatment response, particularly when evaluated through three-dimensional CT volumetry, remains ambiguous. This study investigates whether pretreatment NLR and treatment-related changes in NLR correlate with volumetric tumor response to platinum-based neoadjuvant chemotherapy (NACT). Adult patients with histologically confirmed NSCLC who received platinum-based NACT and had evaluable pre- and posttreatment CT imaging were included in the analysis. Tumor response was defined as a ≥30% reduction using three-dimensional CT volumetry. We analyzed pretreatment NLR, posttreatment NLR, changes in NLR (ΔNLR), and the NLR ratio. To enhance statistical stability, additional analyses utilized log-transformed NLR values. Associations were examined through non-parametric tests, correlation analysis, and both univariable and multivariable logistic regression models adjusted for age, sex, histology, disease stage, performance status, and baseline tumor volume. A total of 70 patients were included, with 33 (47.1%) achieving a volumetric response. NLR decreased during treatment (median ΔNLR ═ −0.42), yet no significant differences were noted between responders and non-responders regarding pretreatment NLR (*P ═* 0.773), posttreatment NLR (*P ═* 0.920), ΔNLR (*P ═* 0.514), or NLR ratio (*P ═* 0.630). None of the NLR-derived parameters showed significant associations with treatment response in univariable or multivariable models, including those utilizing log-transformed variables. Furthermore, the NLR ratio exhibited substantial instability, reflected in wide confidence intervals. In conclusion, within this cohort, neither pretreatment nor dynamic NLR parameters were significantly associated with CT-based volumetric tumor response following platinum-based NACT.

## Introduction

Lung cancer is classified into two primary histological categories: small cell lung cancer (SCLC) and non-small cell lung cancer (NSCLC). NSCLC accounts for nearly 85% of lung cancer cases and includes several histological subtypes, predominantly adenocarcinoma and squamous cell carcinoma [1].

The prognostic significance of the neutrophil-to-lymphocyte ratio (NLR) in NSCLC has been extensively studied, with multiple investigations confirming its reliability as a prognostic biomarker in both early and advanced stages [2, 3]. The NLR serves as an indicator of the host’s inflammatory status [4]. Chronic inflammation fosters tumor development by promoting cellular survival, proliferation, and angiogenesis throughout tumor initiation and progression [5]. Neutrophils contribute to a pro-tumorigenic microenvironment through various mechanisms, such as secreting proteases and growth factors that facilitate tumor invasion and metastasis, promoting angiogenesis, and suppressing effective adaptive anti-tumor immune responses [6]. Conversely, lymphocytes are essential for antitumor immunity, directly targeting malignant cells and helping to control tumor growth across various cancer types [7, 8]. The NLR reflects the balance between tumor-promoting and tumor-suppressive immune mechanisms. Its advantages as a prognostic marker include wide availability and cost-effectiveness [9].

Recent studies have increasingly linked NLR to imaging-based measures of tumor burden, providing a biological rationale for its association with treatment response in lung cancer [10–13].

This retrospective study analyzed data from patients treated in 2025 to evaluate pretreatment NLR as a biomarker for volumetric response to platinum-based neoadjuvant chemotherapy (NACT) in NSCLC.

## Materials and methods

### Study design and setting

This retrospective single-center study aimed to evaluate the predictive value of pretreatment NLR and NLR-derived dynamic parameters, including post-treatment NLR, change in neutrophil-to-lymphocyte ratio (ΔNLR), and NLR ratio, for computed tomography (CT)-based volumetric tumor response following platinum-based NACT in patients with NSCLC.

### Inclusion criteria

Eligible patients were those aged ≥18 years at diagnosis, with histologically confirmed adenocarcinoma or squamous cell lung carcinoma. Additionally, pretreatment and posttreatment contrast CT imaging was required for volumetric assessment of the primary tumor. Participants needed to have completed four cycles of platinum-based NACT and have complete clinical data, including age, sex, and complete blood count obtained before the initiation of NACT and one month after completing the fourth cycle, within a week of follow-up CT after NACT.

### Exclusion criteria

Patients were excluded if they met any of the following criteria: age under 18 years, non-NSCLC histology (including not otherwise specified (NOS) NSCLC or other rare lung malignancies), lack of evaluable imaging studies before and/or after NACT, failure to receive four cycles of platinum-based NACT, incomplete laboratory data, presence of active infection, inflammatory disease, or hematologic disorder at pre and posttreatment blood sampling, concomitant pretreatment use of systemic corticosteroids or immunosuppressive therapy, administration of granulocyte colony-stimulating factor (GCSF), corticosteroids, or blood transfusion during treatment or prior to posttreatment blood sampling. Standard premedication regimens, including antiemetics, were permitted.

### Tumor volume analysis

Tumor volumetry was conducted on contrast-enhanced CT scans using a semi-automated method on the IntelliSpace Portal platform (Philips Healthcare). Two experienced radiologists independently reviewed all CT images and measured tumor volumes, delineating tumor margins while excluding adjacent blood vessels and bronchial structures. Both radiologists were blinded to laboratory data and the temporal sequence of imaging (pretreatment vs. posttreatment CT scans). Interobserver agreement for tumor volumetry was excellent, with an intraclass correlation coefficient (ICC) greater than 0.9, indicating a high level of reproducibility between measurements. A ≥30% reduction in tumor volume was used to define treatment response, consistent with prior work by Hayes et al., who established this threshold as equivalent to Response Evaluation Criteria in Solid Tumors (RECIST)-defined partial response [14].

### Statistical analysis

Continuous variables were assessed for normality using the Shapiro–Wilk test and are presented as medians with interquartile ranges (IQR). Categorical variables are reported as absolute numbers and percentages. Comparisons between two independent groups utilized the Mann–Whitney *U* test for continuous variables and the chi-square test for categorical variables.

NLR was calculated by dividing the absolute neutrophil count by the absolute lymphocyte count obtained from the complete blood count. ΔNLR was defined as the signed difference between posttreatment and pretreatment NLR (ΔNLR = NLRposttreatment – NLRpretreatment), with negative ΔNLR values indicating a decrease in NLR after treatment and positive values indicating an increase. The NLR ratio was calculated as posttreatment NLR divided by pretreatment NLR. Pretreatment NLR, posttreatment NLR, ΔNLR, and the NLR ratio were analyzed both as continuous and categorical variables (≥25% decrease vs. <25% decrease).

Primary analyses included comparisons of pretreatment NLR, posttreatment NLR, ΔNLR, and NLR ratio between responders and non-responders, followed by univariable and multivariable logistic regression models. Secondary sensitivity analyses employed log-transformed NLR variables to account for potential skewness and improve statistical stability. Exploratory subgroup analyses by histological subtype and categorical ≥25% NLR decrease were also conducted. Differences between subgroups were estimated using the Hodges–Lehmann median difference, with corresponding 95% confidence intervals (CIs) reported as measures of effect size precision. Associated *P* values were calculated using the Mann–Whitney *U* test, with results interpreted as exploratory due to limited subgroup sample sizes.

**Table 1:** Clinicopathologic characteristics of the study population by volumetric response

**Characteristic**	**Total (*n* ═ 70)**	**Responders**	**Non-responders**	***P* value**
Age, years, median (IQR)	64 (58–69)	63 (57–68)	65 (59–70)	0.329
Sex:				0.226
Female Male	34 (48.6) 36 (51.4)	13 (39.4) 20 (60.6)	21 (56.8) 16 (43.2)	
Histology:				0.497
Adenocarcinoma Squamous cell carcinoma	38 (54.3) 32 (45.7)	16 (48.5) 17 (51.5)	22 (59.5) 15 (40.5)	
Pretreatment NLR, median (IQR)	2.46 (1.89–3.15)	2.80 (1.80–3.13)	2.34 (1.91–3.38)	0.773
Posttreatment NLR, median (IQR)	2.05 (1.46–2.85)	2.32 (1.44–2.77)	1.99 (1.55–2.90)	0.920
ΔNLR, median (IQR)	−0.42 (−0.64 to −0.16)	−0.44 (−0.65 to −0.15)	−0.39 (−0.61 to −0.17)	0.514
NLR ratio, median (IQR)	0.84 (0.78–0.92)	0.82 (0.78–0.91)	0.86 (0.78–0.92)	0.630
Pretreatment tumor volume, median (IQR)	68,203 (37,625–92,080)	68,125 (38,435–103,427)	72,349 (37,355–90,961)	0.981

**Abbreviations:** IQR, interquartile range; NLR, neutrophil-to-lymphocyte ratio; ΔNLR, change in neutrophil-to-lymphocyte ratio.

Spearman’s rank correlation coefficient assessed the association between pretreatment tumor volume and NLR-derived parameters.

The predictive value of NLR parameters for volumetric tumor response was evaluated using univariable logistic regression analysis. Subsequently, multivariable logistic regression models were constructed to determine whether NLR-derived parameters were independently associated with tumor response after adjusting for age, sex, histological subtype, disease stage, performance status, and baseline tumor volume. Results are presented as odds ratios (ORs) with 95% CIs. Both raw and log-transformed NLR variables were included in the regression models.

All statistical tests were two-sided, and a *P* value < 0.05 was deemed statistically significant. Statistical analyses were performed using Statistical Package for the Social Sciences (SPSS) software, version 26.

### Ethical statement

This study received approval from the Ethics Committee of the Institute for Pulmonary Diseases of Vojvodina, Sremska Kamenica, Serbia (No. 26-I/10), and written informed consent was obtained from all patients prior to inclusion in the study during follow-up examinations. Given the retrospective design, patient data were anonymized prior to analysis, with all personal identifiers removed and replaced by study-specific codes to ensure confidentiality in accordance with institutional and ethical standards.

## Results

A total of 232 patients were screened for eligibility, resulting in the enrollment of 70 patients. Exclusion criteria led to the removal of 54 patients from the study; 36 had non-NSCLC histology (including 36 with SCLC, 7 with carcinoid tumors, and 11 with NOS), 28 lacked evaluable imaging studies, 17 received fewer than four cycles of NACT, 39 had incomplete laboratory data, 11 received GCSF, and 13 were excluded due to blood transfusions during treatment.

Clinicopathologic characteristics of the study population based on volumetric response are presented in Table 1. Prior to NACT, 51 patients were classified as clinical stage III (41 in stage IIIA and 10 in stage IIIB), while 19 patients were in stage IIA. After NACT, 26 patients remained in stage III (19 in IIIA and 7 in IIIB), 34 were in stage II (13 in IIA and 21 in IIB), and 8 patients were in stage IB. All patients with squamous cell carcinoma and the majority with adenocarcinoma received the cisplatin/gemcitabine NACT protocol, while 4 patients with adenocarcinoma received cisplatin/pemetrexed and 3 received carboplatin/paclitaxel. A clinically significant tumor volumetric response was observed in 33 patients (47.1%), while 37 patients (52.9%) were classified as non-responders. The median age of the overall cohort was 64 years (IQR 58–69). No statistically significant age difference was noted between those who achieved a volumetric response and those who did not (median 63 vs. 65 years, *P ═* 0.329). In the study, 51.4% (36/70) of patients were male. Among responders, 13 patients (39.4%) were female and 20 (60.6%) were male, whereas among non-responders, 21 patients (56.8%) were female and 16 (43.2%) were male. No significant association between sex and volumetric tumor response was identified (*P ═* 0.226). Adenocarcinoma was diagnosed in 38 patients (54.3%). The distribution of histological subtypes did not differ significantly between responders and non-responders. Among responders, 16 patients (48.5%) had adenocarcinoma, while 17 (51.5%) had squamous cell carcinoma; in contrast, among non-responders, 22 patients (59.5%) had adenocarcinoma and 15 (40.5%) had squamous cell carcinoma. In the overall cohort, a decrease in NLR during NACT was noted. The median posttreatment NLR was 2.05 (IQR 1.46–2.85), compared to a median pretreatment NLR of 2.46 (IQR 1.89–3.15). Dynamic changes in NLR are illustrated in Figure 1. The median change in NLR (ΔNLR) was −0.42, with a median NLR ratio of 0.84, indicating an overall reduction in NLR during treatment. However, no significant differences were observed between responders and non-responders in pretreatment NLR (*P ═* 0.773), posttreatment NLR (*P ═* 0.920), ΔNLR (*P ═* 0.514), or NLR ratio (*P ═* 0.630).

**Figure 1.**
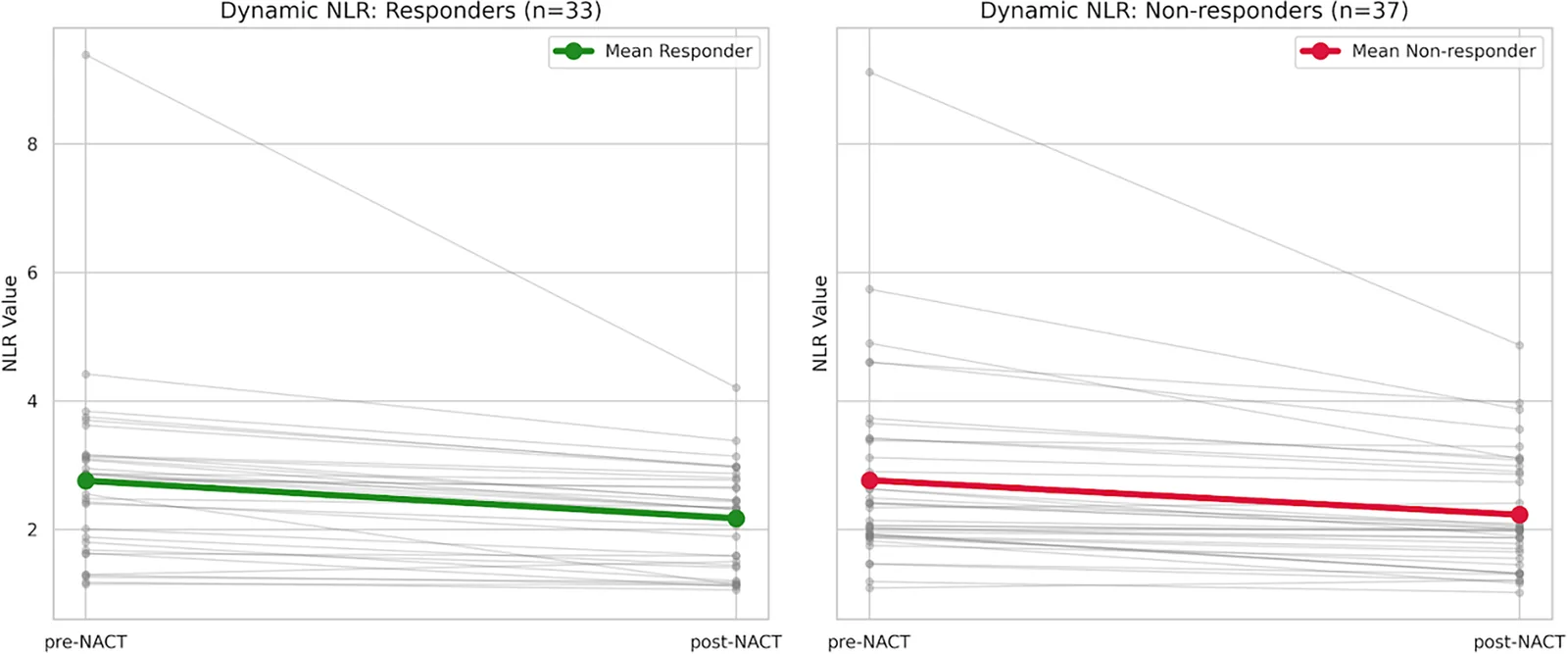
**Dynamic changes in NLR according to volumetric response status.** Individual patient trajectories from pretreatment to posttreatment assessment are shown in gray for responders (left panel, *n* ═ 33) and non-responders (right panel, *n* ═ 37). Colored lines with markers denote the mean NLR in each group (green, responders; red, non-responders). In both groups, NLR values tended to decrease after NACT, consistent with the overall reduction observed in the study cohort. **Abbreviations:** NACT, neoadjuvant chemotherapy; NLR, neutrophil-to-lymphocyte ratio.

The median pretreatment tumor volume for the entire cohort was 68,203 mm^3^ (IQR 37,625–92,080). No significant difference in pretreatment tumor volume was observed between responders (median 68,125 mm^3^; IQR 38,435–103,427) and non-responders (median 72,349 mm^3^; IQR 37,355–90,691) (*P ═* 0.981). Spearman correlation analysis indicated no significant association between pretreatment tumor volume and pretreatment NLR (ρ ═ −0.042, *P ═* 0.733), posttreatment NLR (ρ ═ −0.044, *P ═* 0.716), ΔNLR (ρ ═ 0.044, *P ═* 0.719), or NLR ratio (ρ ═ 0.058, *P ═* 0.634), suggesting that systemic inflammatory status, as measured by NLR, was not directly correlated with tumor burden.

In univariable logistic regression analysis (Table 2), none of the NLR-derived parameters were significantly associated with volumetric tumor response: pretreatment NLR (OR 1.00; 95% CI 0.72–1.37; *P ═* 0.979), posttreatment NLR (OR 0.92; 95% CI 0.53–1.61; *P ═* 0.779), ΔNLR (OR 0.93; 95% CI 0.52–1.66; *P ═* 0.806), and NLR ratio (OR 0.50; 95% CI 0.01–19.85; *P ═* 0.709). Additionally, logistic regression analyses utilizing log-transformed NLR variables did not reveal significant associations with volumetric tumor response. Log-transformed pretreatment NLR (OR 1.00, 95% CI 0.34–2.92, *P ═* 0.997), posttreatment NLR (OR 0.88, 95% CI 0.26–2.95, *P ═* 0.834), and Δlog(NLR) (OR 0.51, 95% CI 0.03–8.34, *P ═* 0.638) were not predictive of response.

**Table 2:** Univariable and multivariable logistic regression analysis of predictors of volumetric tumor response

**Variable**	**Univariable OR** **(95% CI)**	***P* value**	**Multivariable OR** **(95% CI)**	***P* value**
Pretreatment NLR	1.00 (0.72–1.37)	0.979	1.09 (0.76–1.57)	0.627
Posttreatment NLR	0.92 (0.53–1.61)	0.779	0.98 (0.52–1.83)	0.938
ΔNLR	0.93 (0.52–1.66)	0.806	0.73 (0.38–1.41)	0.353
NLR ratio	0.50 (0.01–19.85)	0.709	0.05 (0.0008 – 3.73)	0.177
log (NLR pretreatment)	1.00 (0.34–2.92)	0.997	1.48 (0.42–5.14)	0.541
log (NLR posttreatment)	0.88 (0.26–2.95)	0.834	1.03 (0.26–4.07)	0.966
Δlog (NLR)	0.51 (0.03–8.34)	0.638	0.11 (0.004–2.64)	0.171

**Abbreviations:** CI, confidence interval; NLR, neutrophil-to-lymphocyte ratio; OR, odds ratio; ΔNLR, change in neutrophil-to-lymphocyte ratio.

In fully adjusted multivariable models that included age, sex, histology, disease stage, performance status, and baseline tumor burden, none of the NLR-derived parameters were significantly associated with volumetric tumor response. This consistency was observed in both raw and log-transformed models. Pretreatment NLR (OR 1.09, 95% CI 0.76–1.57, *P ═* 0.627), posttreatment NLR (OR 0.98, 95% CI 0.52–1.83, *P ═* 0.938), and ΔNLR (OR 0.73, 95% CI 0.38–1.41, *P ═* 0.353) did not show significant predictive value. The NLR ratio exhibited substantial instability, with a wide CI (OR 0.05, 95% CI 0.0008–3.73, *P ═* 0.177). Similarly, log-transformed models produced consistent results, with log-transformed pretreatment NLR (OR 1.48, 95% CI 0.42–5.14, *P ═* 0.541), posttreatment NLR (OR 1.03, 95% CI 0.26–4.07, *P ═* 0.966), and Δlog(NLR) (OR 0.11, 95% CI 0.004–2.64, *P ═* 0.171) not significantly associated with response. The wide CIs indicate considerable uncertainty in the estimates.

Exploratory subgroup analyses stratified by histological subtype did not reveal significant associations between any NLR-derived parameters and volumetric response in either adenocarcinoma or squamous cell carcinoma. Detailed subgroup results are presented in Table 3.

**Table 3:** Exploratory subgroup analyses by histological subtype

**Histological subtype**	**Variable**	Effect estimate*	**95% CI**	***P* value**
Adenocarcinoma	Pretreatment NLR	**0.01**	−0.65 to 0.61	0.965
	Posttreatment NLR	**−0.11**	−0.59 to 0.48	0.584
	ΔNLR	**−0.01**	−0.25 to 0.23	0.891
	NLR ratio	**−0.004**	−0.084 to 0.097	0.988
Squamous cell carcinoma	Pretreatment NLR	**0.23**	−0.75 to 0.99	0.706
	Posttreatment NLR	**0.11**	−0.60 to 0.74	0.821
	ΔNLR	**−0.08**	−0.36 to 0.12	0.417
	NLR ratio	**−0.032**	−0.102 to 0.056	0.450

*Effect estimates are expressed as Hodges–Lehmann median differences between responders and non-responders. Negative values indicate that responders exhibit lower values. Abbreviations: CI, confidence interval; NLR, neutrophil-to-lymphocyte ratio; ΔNLR, change in neutrophil-to-lymphocyte ratio.

A decrease in NLR of ≥25% during therapy was defined as a relative reduction of at least 25% from baseline, calculated as (NLRposttreatment − NLRpretreatment) / NLRpretreatment ≤ −0.25. For clarity, this was also expressed as NLRposttreatment ≤ 0.75 × NLRpretreatment. A decrease in NLR of ≥25% was observed in 13 of 70 patients overall, comprising 5 responders and 8 non-responders, with no statistically significant difference between groups (Fisher’s exact test, *P ═* 0.551). In the adjusted logistic regression model, a decrease in NLR of ≥25% was not an independent predictor of response (OR 0.67; 95% CI 0.19–2.35; *P ═* 0.530).

## Discussion

Systemic inflammatory biomarkers have garnered increasing interest as readily available tools for refining risk stratification in NSCLC. Among these, the NLR is one of the most extensively studied, reflecting the balance between systemic inflammatory activation and adaptive immune competence. Hematological parameters play a crucial role in determining treatment tolerance and continuity [15].

Numerous clinical studies have demonstrated that elevated baseline NLR is associated with adverse outcomes in patients with NSCLC. Higher pretreatment NLR has been correlated with reduced survival following surgical intervention for lung cancer, indicating its potential as a prognostic biomarker [16]. Subsequent investigations have confirmed these findings in advanced disease settings, where elevated NLR was independently associated with poorer overall survival [17–19].

Previous studies have suggested that dynamic changes in NLR during anticancer treatment may carry greater clinical relevance than baseline values alone. Park et al. demonstrated that an increase in NLR after concurrent chemoradiotherapy was associated with inferior survival in locally advanced NSCLC, underscoring the prognostic significance of treatment-induced inflammatory shifts [20].

In contrast to the established prognostic role of NLR, evidence regarding its predictive value for radiological response remains limited and inconsistent. Andayani and Arliny found that patients with pretreatment NLR lower than 4.33 were more likely to achieve a partial response or stable disease after systemic therapy for NSCLC, with a sensitivity of 86.7% and specificity of 55.5% [21]. In the study by Faot et al., a baseline NLR exceeding 3.75 correlated with progressive disease after two chemotherapy cycles, although this relationship did not persist after four cycles [13]. Similarly, Kristianto et al. reported that an increase in NLR during treatment, rather than pretreatment NLR, was associated with RECIST-defined progression [22]. Volumetric analysis is more sensitive than one-dimensional (diameter-based) measurements in detecting early treatment-related changes, facilitating earlier recognition of both response and non-response to therapy [23]. This was corroborated by Hayes et al., who demonstrated that CT volumetry may offer a more sensitive and prognostically relevant assessment of treatment response compared to RECIST [14].

In the present study, neither pretreatment nor dynamic NLR parameters were associated with CT-based volumetric tumor response following platinum-based NACT in patients with NSCLC. Although a decline in NLR during treatment was noted, this reduction was observed similarly in both responders and non-responders, indicating that systemic inflammatory changes were not related to the extent of tumor shrinkage. Despite selecting NLR parameters as candidate predictors based on prior evidence, no statistically significant or clinically applicable predictive model could be derived from this cohort.

These discrepancies may be attributed to fundamental methodological differences between RECIST criteria and three-dimensional volumetric assessment. Although RECIST provides a standardized and reproducible response evaluation, diameter-based criteria may not fully capture the complex treatment-induced changes in tumor burden, particularly in irregularly shaped lesions or heterogeneous regression patterns. Another notable finding from our study is the lack of correlation between pretreatment tumor volume and NLR-derived parameters. This observation suggests that systemic inflammatory status, as indicated by NLR, does not merely reflect anatomical tumor burden but rather represents broader host-related inflammatory and immune characteristics. Data concerning NLR dynamics in the NACT context remain limited. Most previous studies in NSCLC have focused on baseline or treatment-induced changes in NLR to explore their association with pathological response or long-term survival across various therapeutic modalities, while the relationship with radiological tumor regression has rarely been addressed [17, 24–27].

Several studies have investigated the association between NLR and positron emission tomography/computed tomography (PET/CT)-derived volumetric parameters, such as metabolic tumor volume and total lesion glycolysis. In these studies, elevated pretreatment NLR has been linked to higher metabolic tumor burden and poorer clinical outcomes in patients with NSCLC [10, 12, 28, 29]. However, PET-derived metabolic parameters primarily reflect tumor metabolic activity rather than anatomical tumor size and are therefore not directly comparable to CT-based volumetric measurements. In this context, the current analysis provides complementary information regarding the relationship between systemic inflammation and anatomical tumor response assessed through CT volumetry.

From a clinical standpoint, our findings suggest that NLR-derived parameters should not be utilized as standalone biomarkers for predicting volumetric tumor regression in patients undergoing NACT for NSCLC. The results also underscore the necessity of distinguishing between prognostic and predictive biomarkers.

This study has several strengths. The cohort was homogeneous, comprising only patients treated with the platinum-based NACT regimen, which minimizes treatment-related variability. Tumor response was evaluated using three-dimensional volumetric analysis, which offers a more accurate representation of tumor burden than conventional diameter-based measurements. NLR-derived parameters were examined, allowing for a detailed assessment of systemic inflammatory changes during treatment. Major conditions known to influence leukocyte counts were minimized through predefined exclusion criteria, enhancing the reliability of NLR as a biomarker.

However, several limitations should be acknowledged. This was a retrospective single-center study with a relatively limited sample size. Exclusions due to missing imaging or laboratory data may affect generalizability. Subgroup analyses by histological subtype were exploratory and should be interpreted cautiously due to the small number of patients in each subgroup. Molecular tumor characteristics and other established predictive biomarkers were not available for analysis. It is crucial to recognize that the absence of significant predictive value for NLR parameters in our regression models may be partially influenced by the sample size, as the statistical power might have been insufficient to detect subtle predictive associations.

Our findings should be contextualized against several differences from prior studies. First, unlike most reports that rely on RECIST criteria, we employed CT-based three-dimensional tumor volumetry, which may limit direct comparability. Second, our cohort consisted of patients treated with NACT who were potential candidates for surgical resection, whereas many previous studies focused on advanced or metastatic settings or different treatment modalities. Third, the relatively small sample size reduces statistical power and contributes to wide CIs, thereby limiting the identification of robust predictive thresholds.

## Conclusion

In this cohort, pretreatment and dynamic NLR parameters were not significantly associated with CT-based volumetric tumor response following platinum-based NACT. Despite an overall decrease in NLR during treatment, none of the evaluated metrics demonstrated meaningful discriminatory or predictive value, including in fully adjusted models. These findings suggest that NLR alone is unlikely to serve as a reliable biomarker of treatment response in this context.

## Data Availability

The datasets used and/or analyzed during the current study are available from the corresponding author on reasonable request.
